# Percutaneous Kyphoplasty combined with different anti-osteoporosis drugs for the treatment of osteoporotic vertebral compression fractures

**DOI:** 10.12669/pjms.41.4.11569

**Published:** 2025-04

**Authors:** Weiliang Du, Wenshuai Li, Lixing Kang, Linfeng Wang

**Affiliations:** 1 Weiliang Du Department of Orthopedics, Langfang People’s Hospital, 37 Xinhua Road, Langfang, Hebei Province 065000, P.R. China; 2 Wenshuai Li, The Third Hospital of Hebei Medical University, 139 Ziqiang Road, Shijiazhuang, Hebei Province 065000, P.R. China; 3 Lixing Kang Department of Orthopedics, Langfang People’s Hospital, 37 Xinhua Road, Langfang, Hebei Province 065000, P.R. China; 4 Linfeng Wang Department of Spine, The Third Hospital of Hebei Medical University, 139 Ziqiang Road, Shijiazhuang, Hebei Province 065000, P.R. China

**Keywords:** Percutaneous kyphoplasty, Osteoporotic vertebral compression fractures, Anti osteoporosis drugs

## Abstract

**Objective:**

To explore the effectiveness of percutaneous kyphoplasty (PKP) combined with different anti-osteoporosis drugs in the treatment of osteoporotic vertebral compression fractures (OVCF) by assessing bone mineral density, pain, lumbar functional recovery, and incidence of vertebral refractures after the combined therapy.

**Methods:**

In this single-center retrospective study, medical records of 138 patients with OVCF who underwent PKP in the Third Hospital of Hebei Medical University between January 2021 and October 2022 were retrospectively analyzed. Among them, 41 patients treated with calcium and alfacalciferol supplementation after PKP (Group-A), 58 patients treated with calcium and calcitonin after PKP (Group-B), and 39 patients treated with calcium, calcitonin, and alendronate sodium after PKP (Group-C). Bone mineral density, pain, lumbar functional recovery, and incidence of vertebral refractures in the three groups were compared before and after PKP.

**Results:**

At six and 12 months after PKP, bone density T-values of Group-B and Group-C were significantly higher than Group-A (P<0.05). The visual analogue scale (VAS) scores of Group-B and Group-C were significantly lower than Group-A (*P*<0.05). At 12 months after PKP, T-value of bone density in Group-C was significantly higher, while the VAS score was significantly lower than Group-B; At six months after PKP, the Cobb angle in Group-B and Group-C was significantly lower than Group-A (*P*<0.05). At 12 months after PKP, Oswestry disability index (ODI) and Cobb angle of Group-B and Group-C were significantly lower than Group-A, and the lowest in Group-C. The extent of vertebral loss in Group-C was significantly lower than Groups A and B (*P*<0.05).

**Conclusions:**

In the treatment of osteoporotic vertebral compression fractures after percutaneous kyphoplasty, a combination of calcium therapy with calcitonin and alendronate sodium had a positive effect, which may effectively improve bone density, pain, and functional status, and reduce the incidence of vertebral body fractures.

## INTRODUCTION

Osteoporosis is a common systemic bone metabolism disease in the elderly and is characterized by reduced bone mass and destruction of bone microstructure.^1^ Progression of osteoporosis eventually leads to bone fragility, and even minor external force or low-energy damage can cause fractures, including osteoporotic vertebral compression fractures (OVCF).^1^,^2^ Percutaneous kyphoplasty (PKP) treatment is commonly used in clinical practice for OVCF to minimize pain and restore certain daily activity abilities.^3^-^5^ During the PKR surgery, a balloon is introduced into the vertebral body, and bone cement is injected. This may promote fracture reduction and fixation, reduce pain, and enhance mobility.

However, while PKP alone can stabilize the vertebral body, and alleviate the pain, it does not delay the process of bone loss, which can directly affect postoperative rehabilitation and may lead to recurrent fractures.^6^-^8^ Therefore, studies suggest that it is crucial to combine the PKP surgery in patients with osteoporotic vertebral compression fractures with the subsequent anti-osteoporosis treatment to improve the bone mass.^3^,^4^,^9^

At present, a combination of PKP and anti-osteoporosis drugs has been widely used in the treatment of patients with OVCF, and its treatment effect has been confirmed in a large number of clinical and practical cases.^3^ However, the comparative efficiency of various combinations of postoperative anti-osteoporotic drugs is still unclear. In recent years, our hospital has administered different anti-osteoporosis drugs to patients after the PKP surgery. This study aimed to retrospectively review and analyze the treatment status of these patients to provide reference opinions for osteoporosis treatment after the PKP surgery.

## METHODS

**Fig.1:**
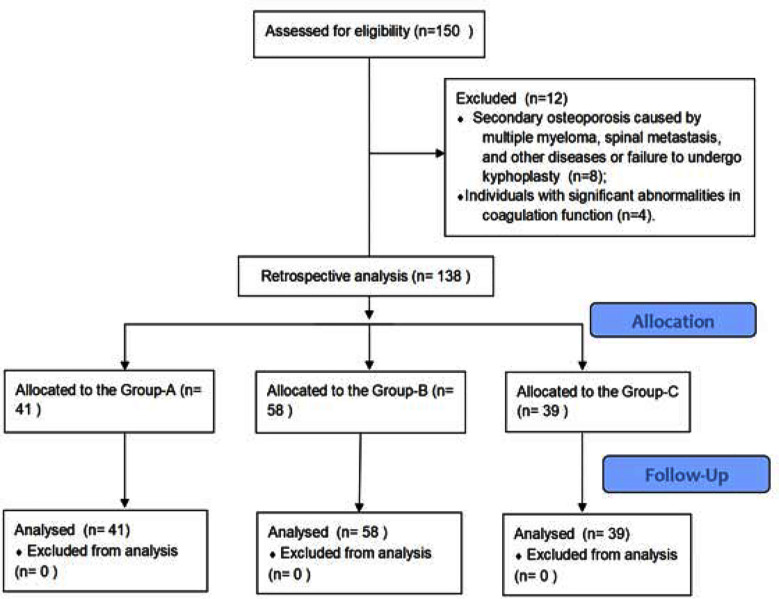
Patient Screening Flow Chart.

In this single-center retrospective study, medical data of 138 patients (74 males and 64 females) who received PKP treatment at the Third Hospital of Hebei Medical University between January 2021 and October 2022 were retrospectively analyzed. Ages of the patients ranged from 54 to 84 years, with an average age of 68.51 ± 6.87 years. According to the treatment records, 41 patients were treated with calcium and Vitamin-D supplementation after the surgery, and were assigned to Group-A, 58 patients were treated with calcium and calcitonin after the surgery, and were assigned to Group-B, and 39 patients received calcium, calcitonin, and alendronate sodium after the surgery, and were assigned to Group-C (Fig.1).

### Ethical Approval:

All procedures conducted in research involving human participants complied with the ethical standards of institutions and/or national research committees, as well as the Helsinki Declaration (revised in 2013). Due to the retrospective nature of the observation and review, the informed consent form was waived. This study was approved by the Medical Ethics Committee (No. 2023-YXLW-016) Date: November 17^th^ 2023.

### Inclusion Criteria:

- Patients with vertebral compression fractures caused by primary osteoporosis^10^ undergoing PKP combined with the use of anti-osteoporosis drugs.
- Complete clinical data and follow-up for more than one year.

### Exclusion criteria:

- Secondary osteoporosis caused by multiple myeloma, spinal metastasis, and other diseases or those who have not undergone kyphoplasty
- Individuals with significant abnormalities in coagulation function.

### PKP surgery:

All patients underwent a bilateral procedure. The patient was placed in the prone position with a solid abdominal cushion. The routine surgical area was disinfected with strong iodine, sterile towels were laid layer-by-layer, and a surgical protective film was applied after alcohol deiodization. The pedicle shadow was located under 3D-C arm fluoroscopy, and local infiltration anesthesia was performed with 0.5% lidocaine from the skin to the periosteum. After successful anesthesia, an incision approximately 0.5 cm long was made, and the specialized puncture needle was placed under the 3D-C arm fluoroscopy at the position of the pedicle shadow. Lateral fluoroscopy was used to confirm that the puncture needle is at the level of the pedicle of the vertebral arch and points towards the center of the vertebral body. With the assistance of 3D-C arm fluoroscopy, the puncture needle was passed through the pedicle of the vertebral arch and extended about 1 cm beyond the posterior edge of the vertebral body.

The upright position showed the needle tip approaching the inner wall of the vertebral arch, and then exiting the puncture needle core. The 3D-C arm machine displayed that the front end of the working channel was located horizontally at the posterior edge of the vertebral body, with an accurate position and direction. The specialized bone drill was rotated along the working channel to the junction area of the anterior and middle 1/4 of the vertebral body. The bone drill was removed, and the expanding balloon was inserted along this channel in 1/3 of the vertebral body. Under the control of the pressure gauge, the balloon was expanded to observe partial recovery of the upper endplate and vertebral height and maintained for five minutes. High viscosity bone cement was mixed and injected into a dedicated bone cement syringe.

The solidification state of bone cement was monitored, and when the cement approached the dough-like stage, a specialized bone-cement syringe was placed along the working channel into 1/3 of the vertebral body. Bone cement was injected from the perspective of the 3D-C arm machine while retreating the syringe to ensure good distribution of bone cement filling. Fluoroscopy confirmed good distribution of bone cement and no signs of infiltration into the spinal canal. The amount of bone cement injected was determined based on the condition of intraoperative fractures, generally 4-5mL for the thoracic vertebrae and 6-8mL for the lumbar vertebrae. After the procedure, nonsteroidal anti-inflammatory drugs or weak opioids was used to control pain in the early postoperative period. After 24-48 hours postoperatively, patients are gradually allowed to get out of bed and move around under the guidance of medical staff. Elastic stockings or intermittent pneumatic compression devices were used to prevent deep vein thrombosis. At 2-6 weeks postoperatively, patients were instructed to perform spinal extension and core muscle group training. After six weeks postoperatively, the intensity of spinal stretching and core muscle training was increased (such as yoga, Chinese tai chi) with low-intensity aerobic exercise (such as walking, swimming). Medications were started the day after surgery.

### Anti-osteoporosis drug treatment: Group-A:

Calcium carbonate D3 (Wyeth Pharmaceutical Co., Ltd., H10950029), 600 mg/dose, oral, 2 times/day; Alfacalciferol Soft Capsule (Qingdao Zhengda Haier Pharmaceutical Co., Ltd., H19991114), 0.50 μg/dose, oral, once a day for 12 months.

### Group-B:

Calcium carbonate D3 and Alfacalciferol Soft Capsule as described for Group-A; salmon calcitonin nasal spray (Novartis Pharma Schweiz AG, H20140632) at a dose of 50IU per dose once a day for 12 months. **Group-C:** Calcium carbonate D3, Alfacalciferol Soft Capsule and salmon calcitonin nasal spray as described for Group-B; alendronate sodium tablets (Beijing WINSUNNY Pharmaceutical Co., Ltd., H20059029) 10 mg/dose, orally, once a day, for 12 months.

### Outcome measures:

- Bone density measured using dual-energy X-ray absorption.
- Pain, evaluated using visual analogue scale (VAS). The scale allows patients to self-select the appropriate pain level on the scale from 0 (no pain) to 10 (severe pain).
- Recovery of lumbar spinal function, as assessed by Oswestry disability index (ODI), Cobb angle, and vertebral loss. The ODI includes 10 questions, each with six options, with a score of 0-5 points, and a total score of (actual score/5) × actual number of questions answered) × 100%; higher score indicates more severe dysfunction. Cobb angle was measured using X-ray examination, and vertebral loss was measured using computed tomography (CT) examination.
- Occurrence of vertebral fractures during follow-up

### Statistical analysis:

Patient data were entered into Microsoft Excel and analyzed using SPSS version 20 (IBM Corp., Armonk, NY, USA) and PRISM8.0 software (GraphPad, San Diego, USA). Continuous variables were reported as mean and standard deviation. ANOVA was used to evaluate the statistical significance of continuous variable differences between the three groups. The Bonferroni post-hoc test was used for paired comparisons. Categorical variables were reported as frequency and percentage, and chi-square tests were used as appropriate to evaluate the differences between the three groups. P< 0.05 was significant. All reported *p*-values were bilateral.

## RESULTS

**Fig.2:**
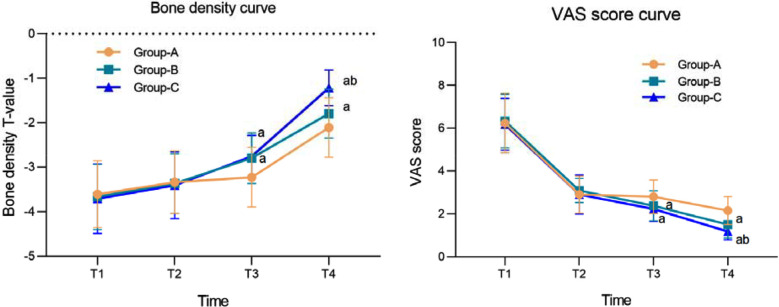
Curve of changes in bone density and VAS score. Compared with Group-A, aP<0.05; Compared with Group-B, b<0.05; T1: preoperative; T2: 3 months after surgery; 6 months after T3 surgery; 12 months after T4 surgery; VAS: Visual Analog Scale.

A total of 138 patients were included in this study: 41 in Group-A, 58 in Group-B, and 39 in Group-C. There was no significant difference in baseline data among the three groups (*P*<0.05) (Table-I). There was no significant difference in the T-value of bone density, and the VAS score between the three Groups-Before and three months after the surgery (*P*>0.05). At six and 12 months after the surgery, bone density T-values of Groups-B and C were significantly higher than those of Group-A, while VAS scores of Groups-B and C were significantly lower than those of Group-A (*P*<0.05). At 12 months after surgery, the T-value of bone density in Group-C was significantly higher, and VAS scores significantly lower than in Group-B (Fig.2).

**Table-I:** Comparison of baseline data among three groups.

Group	Gender (Male/Female)	Age (Year)	BMI (kg/m²)	Fracture site	
				Thoracic vertebra	Lumbar vertebra
Group-A (n=43)	19/24	68.67±6.76	22.64±2.92	19 (44.19)	24 (55.81)
Group-B (n=45)	23/22	67.91±7.34	21.58±3.18	16 (3.56)	29 (64.44)
Group-C (n=44)	26/18	70.25±6.78	21.58±2.69	15 (34.09)	29 (65.91)
*χ^2^/F*	1.939	1.299	1.905	1.098	
*P*	0.379	0.276	0.153	0.577	

**Fig.3:**
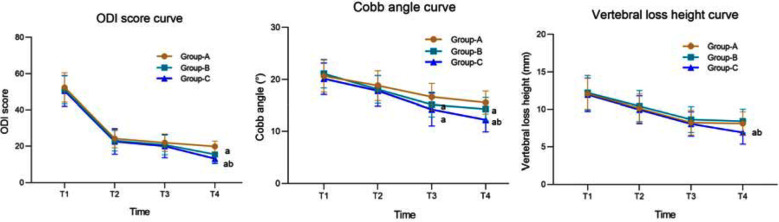
Curve of changes in ODI, Cobb angle, and vertebral loss height. Compared with Group-A, aP<0.05; Compared with Group-B, b<0.05; T1: preoperative; T2: 3 months after surgery; 6 months after T3 surgery; 12 months after T4 surgery; ODI: Oswestry disability index.

There were no significant differences in ODI, Cobb angle, and the extent of vertebral loss in the three groups before and three months after the surgery (*P*>0.05). Six months after the surgery, ODI and vertebral loss were comparable in all groups (*P*>0.05), but the Cobb angle in Groups-B and C was significantly lower than that in Group-A (*P*<0.05). On 12 months follow up, ODI and Cobb angle of Groups-B and C were significantly lower than those of Group-A, and were the lowest in Group-C. The extent of vertebral loss in Group-C 12 months after the surgery was significantly lower than that in Groups A and B (*P*<0.05) (Fig.3). During the follow-up period, one patient in Group-A experienced vertebral fracture six months after the surgery, and one patient in Group-B experienced vertebral fracture 12 months after the treatment, while no vertebral fracture occurred in Group-C.

## DISCUSSION

In the treatment of OVCF after PKP, the comparative efficiency of various combinations of postoperative anti-osteoporotic drugs is still unclear. This study retrospectively compared and analyzed the effects of three anti-osteoporosis regimens on the postoperative treatment of OVCF patients who underwent PKP. Our results show that a combined regimen of calcium, alfacalciferol, calcitonin and alendronate sodium has a good effect, and can effectively improve bone density, reduce pain, promote lumbar functional recovery, and reduce the incidence of vertebral body fractures. Based on the findings of this study, it may provide a reference for the treatment of OVCF after PKP surgery in clinical practice.

Calcium supplementation can fully supplement calcium and promote intestinal absorption of calcium ions, improving bone mineralization and bone mass.^11^ Alfacalciferol is a synthetic vitamin-D compound with functions similar to those of parathyroid hormones. Oral administration of alfacalciferol can promote the increase of 1,25-dihydroxyvitamin-D3 levels in blood circulation, reduce calcium and phosphorus consumption, and promote an increase in calcium and phosphorus levels, thus promoting bone formation.^12^ Calcitonin is a naturally produced peptide that is released from the parathyroid gland, and binds to osteoclasts, inhibiting them from inducing bone resorption.^13^ Studies demonstrate the effectiveness of calcitonin in treating vertebral compression fractures in osteoporosis patients.^14^,^15^ Alendronate sodium is a bone metabolism regulator that interacts with hydroxyapatite in the bone to inhibit the absorption and differentiation of osteoclasts, promote apoptosis, reduce bone loss, and thus reduce bone resorption.^16^,^17^

Dai et al reported that PKP combined with calcitriol and calcium medications could relieve pain and spinal function in patients with traumatic nonosteoporotic vertebral compression fractures.^18^ In the current study, our results confirmed that combined regimen of calcium therapy, alfacalciferol, calcitonin and alendronate sodium have a synergistic effect, achieving good effects in supplementing calcium therapy, improving bone metabolism, increasing bone density, promoting smooth fracture healing, effectively reducing pain, and enabling effective functional recovery of OVCF patients after PKP.^19^,^20^ Additionally, the Cobb angle was significantly improved in Group-B and Group-C. It is believed that after surgery, the patient walks upright, the center of gravity of the upper body is improved, and at the same time, it has a certain effect on the bone formation in the front and back of the vertebral body, which further improves the Cobb angle. Furthermore, the drugs in groups B and C can reduce bone loss, have higher bone density, and have better improvement effects.^21^

Based on the above analysis, the results of our study suggest that anti-osteoporosis regimen of calcium-based combination of calcitonin and alendronate sodium can be efficiently implemented in OVCF patients who undergo PKP surgery. Clinicians should ensure that patients are guided to adhere to standardized anti-osteoporosis medications to further improve clinical efficacy, and bone density, reduce pain levels, and promote effective recovery of lumbar spine function.

### Limitations:

First, this was a single-center retrospective study, which may lead to selection bias. Second, although we attempted to minimize confounding factors in this study, there may have been unmeasurable variables and residual confounding factors in the results. Third, there are few observational indicators, especially laboratory indicators. Fourth, the cost of treatment, the adverse effects of the treatment for each group, and the effects of the comorbidities on outcomes were not studied in this study. Last, the postoperative follow-up period was only 12 months, and a longer follow-up time is needed to verify the results of this study. Finally, a longer follow-up is needed to observe the long-term outcomes and prognosis of patients in the three groups. Future high-quality research is needed to verify our conclusions.

## CONCLUSION

A combination of calcium therapy, alfacalciferol, calcitonin and alendronate sodium has a positive effect in the anti-osteoporosis treatment of patients with OVCF after the PKP surgery. This treatment regimen helps to effectively improve bone density, pain, and functional status, and reduce the incidence of vertebral body fractures.

### Authors’ contributions:

WD: Study design, literature search, and manuscript writing.

WL, LK and LW: Data collection, data analysis and interpretation, Critical Review.

WD: Was involved in the manuscript revision and validation.

All authors have read and approved the final manuscript and are accountable le for the integrity of the study.
